# Learning and memory in zebrafish larvae

**DOI:** 10.3389/fncir.2013.00126

**Published:** 2013-08-02

**Authors:** Adam C. Roberts, Brent R. Bill, David L. Glanzman

**Affiliations:** ^1^Department of Integrative Biology and Physiology, University of California at Los AngelesLos Angeles, CA, USA; ^2^Center for Autism Research and Program in Neurobehavioral Genetics, David Geffen School of Medicine, Semel Institute for Neuroscience and Human Behavior, University of California at Los AngelesLos Angeles, CA, USA; ^3^Department of Psychiatry, David Geffen School of Medicine, University of California at Los AngelesLos Angeles, CA, USA; ^4^Department of Neurobiology, David Geffen School of Medicine, University of California at Los AngelesLos Angeles, CA, USA; ^5^Integrative Center for Learning and Memory, David Geffen School of Medicine, Brain Research Institute, University of California at Los AngelesLos Angeles, CA, USA

**Keywords:** zebrafish, learning, memory, habituation, NMDA receptor

## Abstract

Larval zebrafish possess several experimental advantages for investigating the molecular and neural bases of learning and memory. Despite this, neuroscientists have only recently begun to use these animals to study memory. However, in a relatively short period of time a number of forms of learning have been described in zebrafish larvae, and significant progress has been made toward their understanding. Here we provide a comprehensive review of this progress; we also describe several promising new experimental technologies currently being used in larval zebrafish that are likely to contribute major insights into the processes that underlie learning and memory.

## Introduction

Even relatively simple instances of learning in vertebrates can involve complex interactions of hundreds of molecules, each with distinct spatial and temporal kinetics, as well as neural circuits containing hundreds to thousands of neurons, and thousands to tens of thousands of synapses, which must first be identified and then monitored over time. A proven strategy for reducing this daunting complexity to a manageable level has been to study forms of learning and memory that involve restricted neural circuits. The efficacy of such a reductionist approach has been convincingly demonstrated by investigators of invertebrate learning and memory during the past several decades (Byrne and Kandel, 1996; Dubnau and Tully, 1998; Rankin, 2002; Roberts and Glanzman, 2003; Menzel, 2012). Reductionist neurobiological approaches toward understanding learning in vertebrates have been generally impeded by the enormous size and complexity of the vertebrate brain, especially the mammalian brain. One vertebrate that possesses a nervous system that may be better suited to reductionist analyses of behavior, however, is the zebrafish, *Danio rerio*. Zebrafish display a rich repertoire of behaviors, including associative learning (Norton and Bally-Cuif, 2010; Sison and Gerlai, 2010; Aizenberg and Schuman, 2011; Valente et al., 2012), social learning (Zala and Määttänen, 2013), and shoaling, a type of group behavior (Engeszer et al., 2007). Importantly, they also exhibit simple behaviors that appear to be mediated by relatively simply neural circuits (Kimmel et al., 1974; O'Malley et al., 1996; Easter and Nicola, 1997; Liu and Fetcho, 1999; Eaton et al., 2001; Roeser and Baier, 2003; Gahtan et al., 2005; Burgess and Granato, 2007b; Orger et al., 2008). Furthermore, zebrafish have other qualities that facilitate biological analyses of behavior. For example, because they readily absorb chemicals from water, drugs can be rapidly applied to zebrafish simply by immersing the fish in drug-containing water, which greatly simplifies pharmacological manipulation (Goldsmith, 2004). Undoubtedly one of the most attractive properties of the zebrafish as a model vertebrate organism for the study of behavior, however, is its ease of genetic manipulability. Indeed, the zebrafish approaches such invertebrate models as *Drosophila* and *C. elegans* with respect to the number of forward (Gaiano et al., 1996; Haffter et al., 1996; Schier et al., 1996; Kotani et al., 2006; Sivasubbu et al., 2006) and reverse genetic approaches to which it is amenable (Nasevicius and Ekker, 2000; Wienholds et al., 2003; Guo, 2004; Doyon et al., 2008; Meng et al., 2008; Dong et al., 2009; Bedell et al., 2012; Cade et al., 2012; Dahlem et al., 2012; Hwang et al., 2013). A major advance in genetic manipulation in zebrafish has been the recent development of an effective GAL4/Upstream Activating Sequence (GAL4/UAS) system for use in zebrafish (Asakawa and Kawakami, 2008; Halpern et al., 2008). This system, described in more detail below, enables researchers to target the expression of genes to specific cells. In particular, the GAL4/UAS system has been used to express the genes, such as green fluorescent protein (GFP), as well as of optical probes, such as channelrhodopsin and halorhodopsin, in specific groups of neurons in the zebrafish CNS (Scott et al., 2007; Scott, 2009; Wyart et al., 2009; Warp et al., 2012). These innovations allow neuroscientists to visually identify behaviorally relevant neural circuits in the zebrafish brain and spinal cord, and to optically monitor the functional activity of these circuits. The technical challenges posed by such studies are greatly reduced in the zebrafish by a remarkable feature of its larval form, namely that it is translucent; this feature permits optical investigations of neuronal structure and activity in the intact, and in some instances, behaving animal. Identified neurons can be photoactivated or inhibited in the intact zebrafish larva, and the effect of this optical manipulation of neuronal activity on behavior examined (see Baier and Scott, 2009; Friedrich et al., 2010). Optical manipulation of neural activity is also presently feasible in mammals (e.g., Yizhar et al., 2011) of course; but the specificity of the resulting pattern of activity, as well as its behavioral consequences, is significantly less restricted, and therefore less mechanistically informative, than in the larval zebrafish.

**Figure 1:**
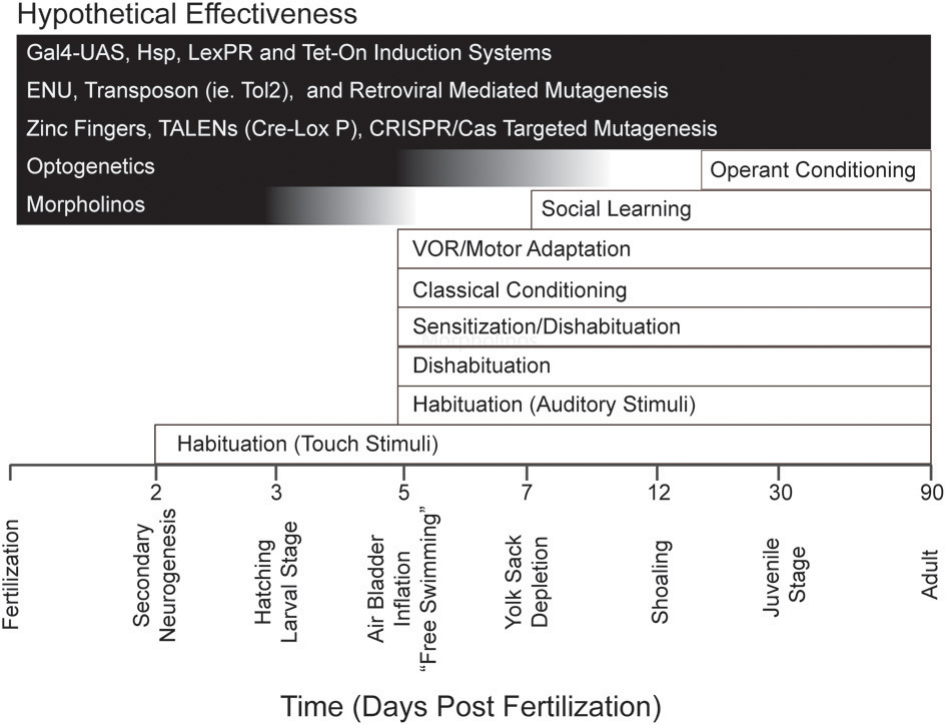
**Repertoire of behavior and learning exhibited by zebrafish during development together with major experimental techniques and their effective age of use**. The timeline at the bottom of the figure indicates important developmental milestones in zebrafish. Note that several forms of learning have been identified as early as 5 dpf, an age at which such powerful experimental techniques as optogenetics are still effective. Note, also, that there are many molecular tools (for example, the GAL4-UAS system; see Halpern et al., 2008) that are effective at all developmental stages in zebrafish.

The readiness with which zebrafish larvae lend themselves to optogenetics and other molecular tools (each with its own distinct efficacy across development), together with the general experimental advantages of zebrafish for reductionist analyses of behavior possessing different ontogenies (Figure 1), might be expected to excite interest among neuroscientists focused on learning and memory. However, to date the cognitive capabilities of larval zebrafish have been relatively unexplored. Here, we will review the various forms of learning and memory shown in these animals. We will also discuss what is currently known regarding the cellular and molecular mechanisms that underlie these forms of learning and memory. Finally, we will discuss potential future directions in learning and memory research in zebrafish larvae.

## Types of learning and memory in larval zebrafish

### Habituation

Habituation is a non-associative form of learning during which the response of an animal to repeated presentations of a stimulus of fixed intensity or strength gradually declines; furthermore, this decline is not due to sensory adaptation, motor fati, or injury (Thompson and Spencer, 1966; Rankin et al., 2009). Despite habituation's simplicity and apparent ubiquity, at present we lack a comprehensive neurobiological understanding of this form of learning (Glanzman, 2009).

**Figure 2:**
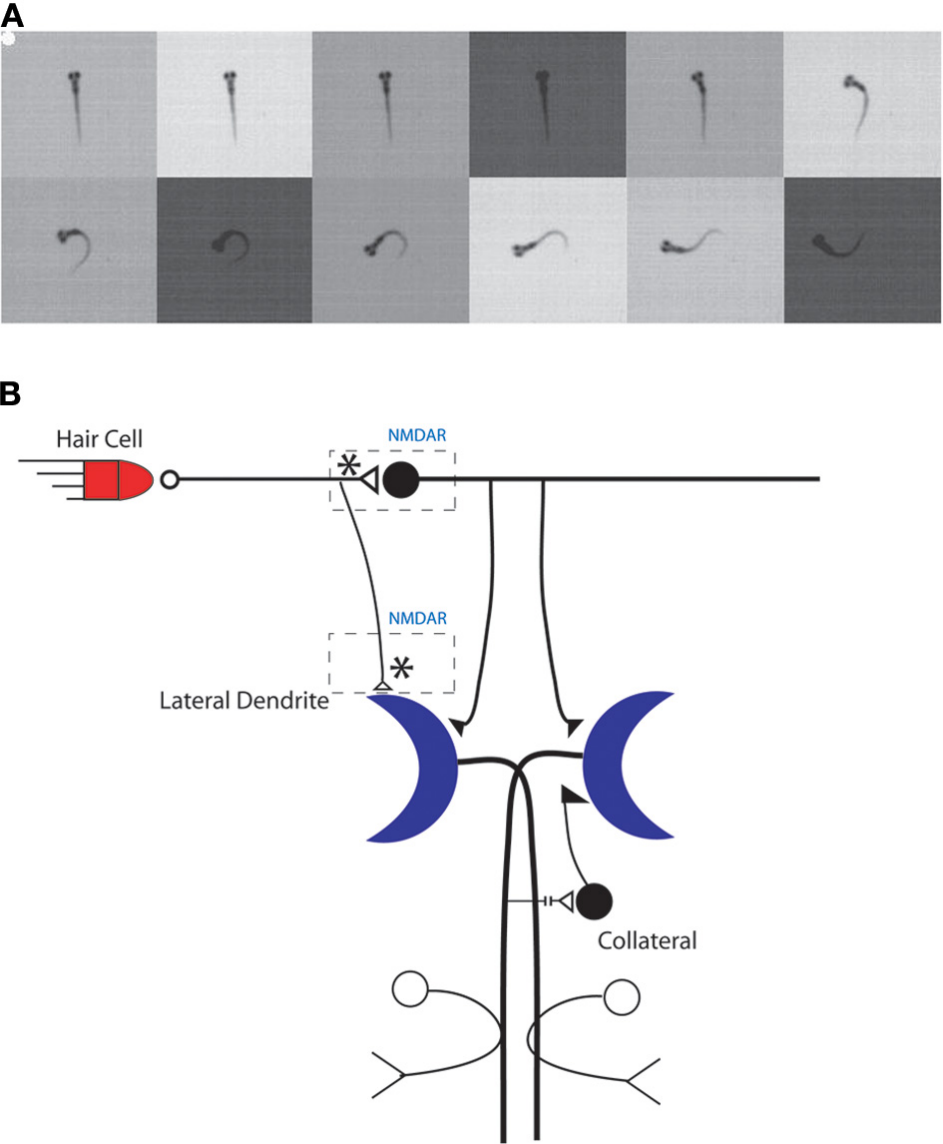
**The C-start reflex in larval zebrafish is mediated by the Mauthner neuron-mediated circuit. (A)** An example of a larval zebrafish C-start reflex in response to an auditory/vibrational stimulus. The initiation of the C-start reflex is marked by a white dot and images were recorded every 1 ms. Frames are shown every 2 ms for illustration purposes. **(B)** Model of the Mauthner neuron circuitry. Potential sites of NMDAR-dependent plasticity are indicated by asterisks. Adapted with permission from Roberts et al. (2011).

Teleost fish, including zebrafish, exhibit a simple startle response, the C-start, that is controlled by a bilateral pair of large command neurons, the Mauthner cells, in the fish's hindbrain (Eaton et al., 2001) (Figure 2). The C-start is triggered by an abrupt sensory (auditory, visual, or tactile) stimulus (Eaton et al., 1984; Weiss et al., 2006); it first appears in zebrafish larvae in response to an auditory stimulus at 4 days postfertilization (dpf), and the response begins to exhibit habituation to a repetitious sensory stimulus at about the same time (Eaton et al., 1977). The onset latency of the C-start is rapid (~6 ms) and the resulting behavior of the fish—the bending of the fish into a C shape, from which the response gets its name—is highly stereotyped (Burgess and Granato, 2007b; Issa et al., 2011); its function is to rapidly propel the fish away from potential predators. Zebrafish larvae also exhibit a related escape behavior that has a longer onset latency (~30 ms), is less stereotyped than the C-start, and is mediated by the activity of non-Mauthner cell circuits rather than by the Mauthner cells (Burgess and Granato, 2007b; Issa et al., 2011) (but see Liu and Fetcho, 1999).

**Figure 3:**
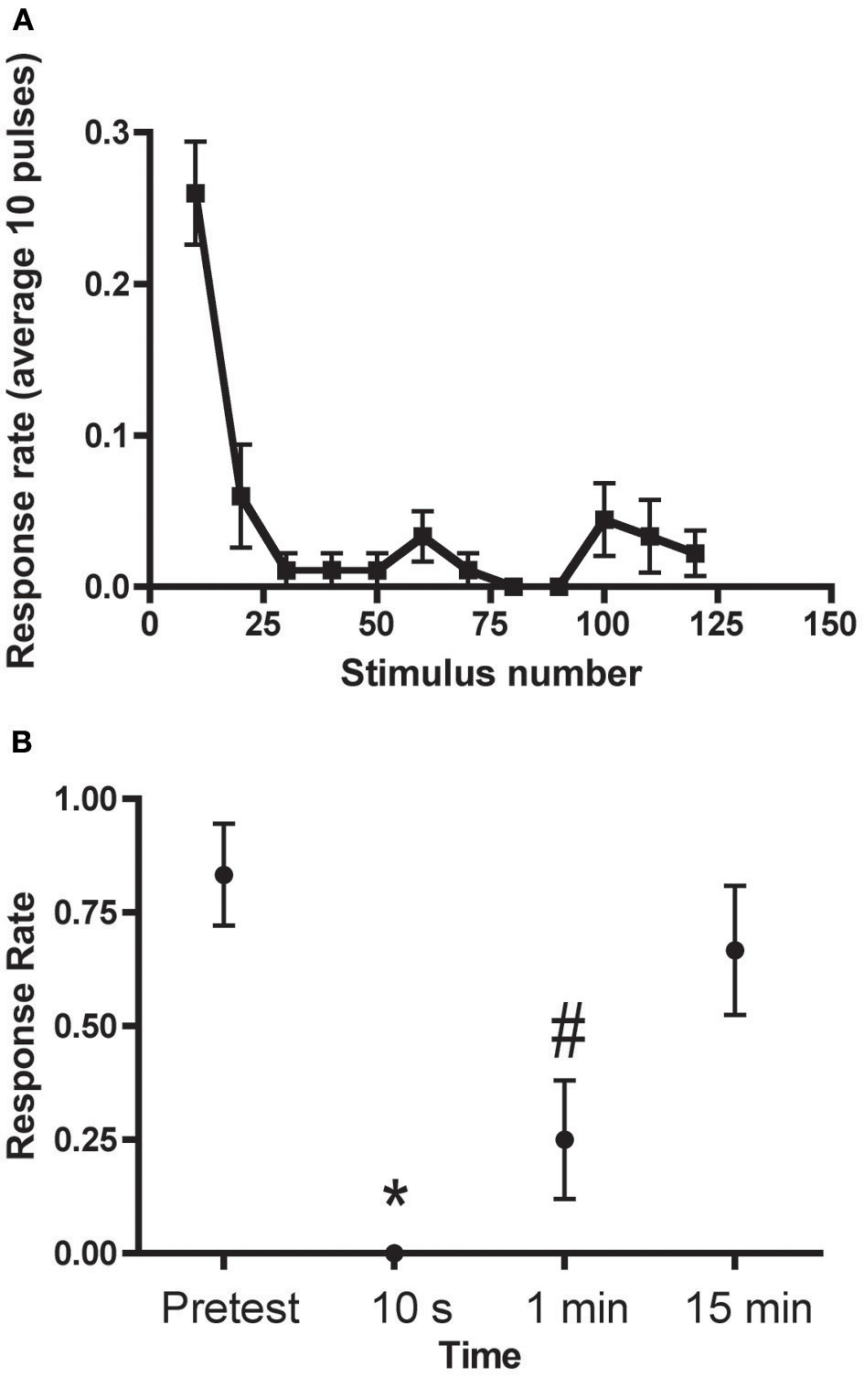
**Rapid Habituation of the C-start (A) C-start responses to 120 pulses delivered at 1 Hz (binned into groups of 10)**. **(B)** Persistence of rapid habituation. The training protocol resulted in short-lived habituation of the C-start, which returned to its original (pretraining) level of responsiveness within 15 min. The asterisk indicates that the 10 s test is significantly different from the pretest and 15 min test; the pound sign indicates that the 1 min test is significantly different from the pretest and the 15 min test. Adapted with permission from Roberts et al. (2011).

Three forms of habituation of the C-start have been described. These forms are induced by different training protocols and are mechanistically distinct. There are two forms of relatively short-lived habituation that we have termed “rapid” and “short-term” (Roberts et al., 2011). Rapid habituation can be induced by massed presentation of 50–120 brief auditory pulses (1 ms in duration, 200 Hz ramp wave, 109 dB), or “pips,” at 1 Hz (Figure 3); the consequent habituation is significant at 1 min after training but the response returns to its initial strength within 3–15 min post-training (Roberts et al., 2011; Wolman et al., 2011). Short-term habituation (STH), which persists for up to 1 h after training, is induced by spaced training, specifically, by 10 blocks of 900 pips (1 Hz) with a 5 min interblock interval (Roberts et al., 2011). Roberts et al. (2011) found that STH of the C-start requires *N*-methyl-d-aspartate receptor (NMDAR) activity, whereas rapid habituation does not. Wolman et al. (2011), however, reported that NMDAR activity was required for rapid habituation as well. (The source of this discrepancy may be the specific NMDAR antagonist used by the two groups; Roberts and colleagues observed that MK801, a non-competitive NMDAR antagonist, used by Wolman and colleagues, did disrupt rapid habituation, whereas dl-2-amino-5-phosphonopentanoic acid (APV), a competitive NMDAR antagonist used in the experiments of Roberts et al., did not alter rapid habituation). Recently, we (Pearce et al., 2012) have shown that the C-start can also undergo long-term habituation (LTH) in larval zebrafish. Here, the fish were stimulated with six spaced blocks of auditory pips; each block comprised spaced 8 trains of pips (120 pips at 1 Hz per train). The spaced training produced significant habituation of the C-start in the larvae that persisted for at least 18 h. Like STH, the induction of LTH depended on the activity of NMDARs; unlike STH, however, LTH depended on macromolecular synthesis, because its induction was disrupted by cold shock, and gene transcription—bathing the fish in the transcriptional inhibitor 5,6-dichlorobenzimidazole 1-β-D-ribofuranoside (DRB) during training blocked LTH. LTH of the C-start in larval zebrafish exhibits a striking mechanistic similarity to LTH of the gill- and siphon-withdrawal reflex in the marine snail *Aplysia* in its requirement for NMDAR activity, translation, and transcription (Ezzeddine and Glanzman, 2003; Esdin et al., 2010).

In response to the sudden extinction of light (“dark flash”) larval zebrafish show another form of escape behavior that differs from the C-start; this response, the O-bend, is characterized by a significantly larger amplitude bend of the fish's body than occurs during the C-start (Burgess and Granato, 2007a). Unlike the C-start, the O-bend is not mediated by the Mauthner cells. Like the C-start, however, the O-bend can undergo LTH as a result of spaced training (120 min of exposure to dark flashes using an interstimulus interval of 15–60 s) (Wolman et al., 2011). LTH of the O-bend persists for up to 24 h and requires protein synthesis, as indicated by its blockage when training is performed in cyclohexamide, a translational inhibitor.

The experiments documenting LTH of escape behaviors in larval zebrafish represent a major advance because they demonstrate, for the first time to our knowledge, that immature zebrafish possess the capacity for long-term memory. Similar to long-term memory for a wide variety of learning tasks in a broad range of organisms, long-term memory in zebrafish larvae is more readily induced by spaced than by massed training (Ebbinghaus, 1964), training, and depends on protein synthesis and gene transcription (Davis and Squire, 1984; Goelet et al., 1986; Yin et al., 1994, 1995; Alberini, 2009; Ardiel and Rankin, 2010).

### Dishabituation and sensitization

Sensitization is a form of non-associative learning in which exposure to an arousing stimulus, commonly one that is painful or noxious, causes response enhancement (Groves and Thompson, 1970). The same stimulus that induces sensitization can typically be used to enhance a habituated response, a phenomenon known as dishabituation. Despite their phenomenological (Thompson and Spencer, 1966; Hawkins et al., 1998) and mechanistic (Antonov et al., 1999) similarities, sensitization and dishabituation are now recognized to be distinct forms of learning (Hawkins et al., 2006; Antonov et al., 2010).

Dishabituation of the rapid escape response in zebrafish larvae (5–7 dpf) has been observed by three laboratories. After habituating the escape response in larvae to auditory pips, Best et al. (2008) produced dishabituation by delivering a brief stimulus of a different sensory modality (a pulse of light) to the fish. Similarly, Wolman et al. (2011) first habituated the C-start in larvae to acoustic stimuli, and then dishabituated it by applying a brief tactile stimulus to the larval head. Using similar methods we have recently succeeded in dishabituating the C-start following LTH of this response.

A short-lived form of cross-modal modulation of the C-start that resembles sensitization (the enhancement of a non-habituated response) has also recently been shown in larval zebrafish. Mu et al. (2012) used auditory pips to evoke the C-start in 5–6 dpf zebrafish. When the sound stimulus was preceded by about 500 ms by a brief (15 ms) pulse of white light (a “flash”), the probability of a C-start being evoked by the subsequent sound stimulus was facilitated; by itself, the flash did not evoke the escape response. Through whole-cell electrophysiological recordings from the Mauthner cell in paralyzed fish embedded in agar, the investigators found that the preceding visual stimulus significantly enhanced the compound synaptic current (CSC) evoked in the Mauthner cell by the auditory pips; the flash alone, however, evoked only a very small CSC. Furthermore, a preceding flash enhanced the biphasic excitatory postsynaptic current (EPSC) evoked in the Mauthner cell by extracellular stimulation of the VIIIth cranial nerve, which transmits auditory information to the brain. The biphasic EPSC contains an early electrical component and a later chemical component; the chemical component is mediated by α-amino-3-hydroxy-5-methyl-4-isoxazolepropionic acid receptors (AMPARs) and NMDARs; both the electrical and chemical components of the EPSC were enhanced by the preceding flash. Mu et al. showed that the enhancement of VIIIth nerve-Mauthner cell synaptic transmission produced by a preceding visual stimulus was mimicked by exogenous application of dopamine and was blocked by antagonists of the D1 dopamine receptor. In support of the idea that the flash causes release of dopamine within the C-start circuit, laser ablation of the GFP-expressing dopaminergic neurons in the brains of the larval fish, as well as down-regulation of dopamine synthesis in hypothalamic dopaminergic neurons by knocking down tyrosine hydroxylase (the enzyme that converts L-tyrosine to L-DOPA) or two transcription factors required for the development of dopaminergic neurons with morpholino oligonucleotides (Mu et al., 2012) reduced the modulation of the auditory-evoked C-start by the preceding flash. Finally, the investigators determined that the visual flash induced bursting activity in dopaminergic neurons in the hypothalamus. It is interesting that the basis of cross-modal enhancement of the auditory-evoked C-start in larval zebrafish is modulatory neuronal actions caused by the release of dopamine within the fish's brain. This scheme is broadly consistent with that for sensitization of the defensive withdrawal reflex in *Aplysia*, which results from modulatory actions on sensorimotor pathways within the snail of another monoamine, serotonin; serotonin's release, in turn, is triggered by noxious stimulation (Brunelli et al., 1976; Castellucci and Kandel, 1976; Kandel and Schwartz, 1982).

The enhancing action of the flash on the sound-elicited C-start in zebrafish larvae appears to be quite brief. It remains to be determined whether more persistent enhancement could be induced in the larvae. Possibly, the briefness of the flash-induced modulation of the escape response may reflects the developmental immaturity of monoaminergic neurotransmission within the CNS of larval fish. In support of this idea, Buske and Gerlai (2012) report that levels of dopamine and serotonin increase dramatically in zebrafish around 10–12 dpf (see below).

Drug-induced sensitization of locomotor activity has been shown in both larval (Petzold et al., 2009) and adult (Blaser et al., 2010) zebrafish. Petzold et al. (2009) observed that the locomotor activity of larval (5–6 dpf, but not 4 dpf) zebrafish was enhanced by nicotine and that re-exposure to the drug sensitized the nicotine response. Interestingly, administration of APV together with nicotine blocked the sensitization. Therefore, NMDAR activity appears to mediate at least some forms of habituation and sensitization. Blaser et al. (2010) examined the effects of repeated exposure to ethanol on locomotor activity in adult zebrafish. They observed sensitization of ethanol-induced hyperactivity in the fish; furthermore, the sensitization was context-specific: fish given a second exposure to ethanol in the same context in which they received their first exposure exhibited enhanced locomotor hyperactivity, whereas fish re-exposed to ethanol in a different context did not show sensitization. (Note that the fish did not classically condition to the context, because their locomotor activity did not increase when they were re-exposed to the original context in the absence of ethanol.) Context specificity of drug-induced sensitization of locomotor activity remains to be shown in larval zebrafish.

### Classical conditioning

Classical conditioning, first described by Pavlov (1927), is the ability of an animal to associate a neutral stimulus (the conditioned stimulus or CS) with a reinforcing stimulus (the unconditioned stimulus or US). As the result of the paired delivery of a CS and a US, the CS acquires the ability to predict the occurrence of the US and, consequently, the animal's response to the CS (the conditioned response or CR) comes to resemble its response to the US (the unconditioned response or UCR). Classical conditioning is the most basic form of associative learning; consequently, understanding its biological basis is a major goal of behavioral neuroscientists.

Adult teleost fish classically condition readily (Agranoff and Davis, 1968; Flood et al., 1976; Amiro and Bitterman, 1980; Mattioli et al., 1998; Eisenberg et al., 2003; Salas et al., 2006; Yoshida and Kondo, 2012), and there have been several published reports of classical conditioning in adult zebrafish (Braubach et al., 2009; Agetsuma et al., 2010; Karnik and Gerlai, 2012; Aoki et al., 2013). To date, there have been just two reports of successful classical conditioning in larval to juvenile zebrafish. In one successful study, Aizenberg and Schuman (2011) trained 6-to-8-day-old larval zebrafish to associate a moving spot of light (the CS) with a touch to the side of the body (the US). The fish were partially restrained in agarose during the experiments such that their tails were free to move. The CR was enhanced movement of the tail in response to the CS. Aizenberg and Schuman also measured changes in intracellular Ca^2+^ in cerebellar neurons in the restrained fish during the experiments. They observed that prior to training the CS and the US activated partially distinct populations of cerebellar neurons prior to conditioning; as a consequence of learning, the number of CS-activated neurons in the cerebellum was increased. Laser-ablation of the cerebellum immediately after the first training trial prevented conditioning, whereas cerebellar ablation after the last training trial impaired extinction of the CR. Interestingly, ablating the cerebellum after training, although it altered extinction, did not affect memory retention, which suggests that the memory for the CR is stored outside the cerebellum.

In the second demonstration of classical conditioning Valente et al. (2012) trained fish to associate a visual pattern projected onto an LCD screen below half of the tank of water in which the fish freely swam (the CS), to a whole-tank electric shock (the US). The experimenters measured the turns away from the side of the tank to which the CS was delivered as the CR. The zebrafish did not exhibit significant evidence of learning on this task until 4 weeks of age (~late larval stage or early juvenile stage), after which their learning steadily improved, reaching an adult level at 6 weeks of age. Valente and colleagues also used a modification of this learning task to attempt to train larval zebrafish. In this modification the CS was a visual stimulus projected from below the fish, which were restrained in agarose, and the US was either an electric shock applied to the head of the fish, or a tap delivered to the fish's ear. However, the training did not produce evidence of learning in 7-day-old larvae.

### Motor learning

A type of vertebrate motor learning that has both formal and mechanistic similarities to classical conditioning is the vestibulo-ocular reflex (VOR) (Lac et al., 1995; Cohen et al., 2004). The VOR is a reflexive eye movement in which vestibular signals are used to generate compensatory eye movements in the opposite direction from head movements; its function is to stabilize retinal images. Calibration of the VOR requires motor learning; when head movements are consistently paired with the undesirable motion of the retinal image, learning occurs and the gain of the reflex is changed to reduce the image motion. Learning in the VOR depends on the cerebellum; furthermore, the cellular mechanisms that mediate this form of learning resemble those that mediate classical conditioning of the eyeblink response (Lac et al., 1995). Adult teleost fish, including adult zebrafish, exhibit a robust VOR (Graf and Baker, 1983; Pastor et al., 1994; Marsh and Baker, 1997). Initially, it was reported that larval zebrafish exhibit angular VORs (VORs evoked by stimulation of the semicircular canals) by 96 h postfertilization (hpf) (Easter and Nicola, 1997; Moorman et al., 2002); however, a later study did not find angular VORs in zebrafish until 35 dpf (Beck et al., 2004). Mo et al. (2010) reexamined this issue, and found evidence for an angular VOR in zebrafish as early as 72 hpf; furthermore, this group showed that several lines of mutant fish with defects of the vestibular system exhibited either a loss of VOR or reduced VOR. Mo and colleagues attributed the earlier failure to recognize the VOR in larval zebrafish (Beck et al., 2004) to mistaken attribution of vestibularly mediated eye movements to visually mediated movements. More recently, Bianco et al. (2012) also reported that larval zebrafish possess a VOR.

A recent study used motor learning in paralyzed zebrafish larvae, together with whole brain imaging of activity-dependent changes in intracellular calcium in individual neurons, to show the promise of larval zebrafish as model organisms for cellular investigations of learning (Ahrens et al., 2012). Ahrens and colleagues examined a type of motor adaptation (the optomotor response) related to the VOR. Here, paralyzed, restrained zebrafish larvae were exposed to a moving whole-field visual stimulus that simulated the visual effect in freely swimming fish of being swept backwards by the water flow. In response, the fish initiated motor commands (“fictive swims”) that would have—were they not paralyzed—moved them forward; the purpose of these fictive swims was to stabilize the virtual location of the fish. The motor commands were recorded electrophysiologically from motor neurons in the fish, and these electrical signals were then translated into visual feedback that mimicked the optic flow produced in freely swimming fish by forward movement. The fish used in this study were transgenics that expressed the calcium sensor GCaMP2 (Akerboom et al., 2012) in almost all neurons. By means of two-photon microscopy the investigators were able to optically record neural activity throughout the brain at single-neuron resolution while the fish “behaved” in the virtual reality setup. They observed many neurons in the inferior olive and cerebellum whose firing correlated with motor adaptation by the fish to the visual stimulation. That the activity of these neurons was somehow causally related to the fish's behavior was indicated by the fact that lesioning the inferior olive post-training eliminated the motor adaptation. Although this study was unable to specify the actual cellular mechanism of motor learning, it nonetheless represents an impressive demonstration of the analytic power of the combination of transgenic manipulation and optical recording in the living, intact brain that zebrafish larvae enable.

### Operant conditioning

Operant conditioning, another major form of associative learning, differs from classical conditioning in that the consequences (outcomes) of an animal's voluntary response to a reinforcing stimulus alters the future probability of the animal's responses or behavior; in classical conditioning the animal's (involuntary) responses to the training stimuli are not altered by the behavior's outcomes (Gluck et al., 2014). One operant conditioning paradigm that has been used successfully with fish is avoidance conditioning. In a protocol originally developed for use with the goldfish over 40 years ago (Agranoff and Davis, 1968; Agranoff, 1971), fish must learn to swim to the other side of a shuttle box at the onset of a light to avoid an electric shock. Adult zebrafish condition readily in this protocol (Pradel et al., 1999, 2000; Xu et al., 2007) moreover, the learning depends on NMDARs (Blank et al., 2009). Two studies have used variants of the original shuttle box training protocol to show avoidance conditioning in larval to juvenile zebrafish. Lee et al. (2010) trained three-to-five-week-old fish to avoid the side of a shuttle box illuminated with a red light. They showed that the learning required the habenula—a diencephalic structure involved in the regulation of dopaminergic and serotonergic pathways, and which mediates avoidance learning in mammals (Shumake et al., 2010)—by using genetic technologies to disrupt habenular circuits. Lee and colleagues used a mutant line that expresses the phototoxic fluorescent protein, KillerRed, in forebrain afferents to the habenula; photobleaching KillerRed-expressing neurons by illuminating the larvae with green light damaged the afferents and, when performed prior to behavioral training, prevented acquisition of conditioned avoidance. Interestingly, photobleaching habenular afferents after training did not impair expression of the learning. Lee et al. also used the GAL4/UAS system to express tetanus toxin specifically in habenular neurons. (This toxin prevents neurons from releasing transmitter by cleaving synaptobrevin.) Larvae with habenular expression of tetanus showed deficits in avoidance conditioning, particularly in the later training trails. The study of Lee and colleagues nicely illustrates the potential of zebrafish for investigations of learning and memory involving modern genetic tools. Valente et al. (2012) also used a shuttle box-type protocol to measure the ontogeny of operant conditioning in zebrafish. The zebrafish did not exhibit significant conditioning until 3 weeks of age (~late larval stage), reaching a maximal (adult) level by week 6.

### Social learning

A shoal is a group of fish, typically of the same species and age, that swim together for social reasons. (Shoaling is distinguished from schooling in which fish swim together in tight, synchronized fashion.) It is believed that this social behavior serves, in part, as a protection against predation through increasing the detection of predators and decreasing the probability of individual capture (Peichel, 2004). As first shown by McCann et al. (1971), shoaling preferences in zebrafish have been shown to be at least partly learned. More recently, Engeszer et al. (2004) examined preferences of zebrafish who had been raised from hatching either in isolation, with siblings of the same phenotype, or with siblings of a different phenotype (cross-rearing). The fish in the study were either wild-type (normally striped) or mutant fish lacking melanophore stripes (*nacre* mutants). The fish were tested for social preference when they reached adulthood (≥90 dpf). Social isolates exhibited no preference for either the wild-type or *nacre* phenotypes. However, fish in the other groups preferred the phenotypes they had been raised with, e.g., wild-type fish raised with *nacre* fish from hatching preferred to shoal with *nacre* fish as adults. These results suggest that shoaling preferences are determined, at least partly, by early experience. In a later study (Engeszer et al., 2007) Engeszer and colleagues ascertained the onset of conspecific preferences in zebrafish; they found that zebrafish begin to show conspecific preferences at approximately the post-flexion stage (~12 dpf), and that zebrafish first exhibit significant shoaling preferences as juveniles. Furthermore, shoaling preferences were not plastic; as adults zebrafish preferred to shoal with the phenotypes they were reared with, even if given prolonged (30 days) exposure to the other phenotypes in adulthood. In addition to visual features, olfactory cues have also been shown to be a significant factor in determining shoaling preferences in zebrafish (Gerlach and Lysiak, 2006; Gerlach et al., 2007).

Two papers by Gerlai and colleagues provide some additional support for the notion that shoaling is a learned social behavior in zebrafish. Al-Imari and Gerlai (2008) raised zebrafish singly to adulthood (the experimental fish). Then the experimental fish were given 10 training trials in which they were placed in a four-arm aquatic maze. Beside each arm of the maze was a small tank—the contents of which were visible from the maze arm—one of which contained seven stimulus fish; a red plastic cue card was placed at the end of the maze arm next to the tank containing the stimulus fish. (The location within the maze of the stimulus fish and red cue card was changed from trial to trial.) Another group of fish (unpaired group) was given ten training trails in the maze, but the red cue card and the stimulus fish were placed separately in different arms. (The locations of the cue card and the stimulus fish were also moved from trial to trial.) Following the training the fish were presented with the red cue card alone, and the amount of time the fish spent in proximity to the card was measured. Fish in the paired group spent significantly more time near the red cue card than would be predicted by chance alone, whereas fish in the unpaired group preformed at chance level, i.e., the amount of time the unpaired fish spent in proximity to the card was no more than would predicted by chance given the total area of the maze. This result demonstrates that the zebrafish found the presence of a shoal mate rewarding. A second paper from this laboratory, that of Buske and Gerlai (2012), used high-pressure liquid chromatographic (HPLC) analyses of whole-brain extracts and behavioral measurements of the tendency to shoal to gain insights into potential neurobiological processes underlying the ontogenesis of shoaling in zebrafish. These investigators found, as had others (Engeszer et al., 2007; Buske and Gerlai, 2011), that shoaling-related behavior increased gradually in zebrafish from 10 to 75 dpf. At the approximately the same time there were also significant increases in the brain levels of both dopamine (DA) and serotonin (5-HT). Although this correlation does not prove that the increase in the brain levels of the monoamines underlay the increase in shoaling behavior, it is nonetheless suggestive; dopaminergic and serotonergic processes are known to play prominent roles in many forms of vertebrate and invertebrate learning and memory (e.g., Kandel, 2001; Wise, 2004; Riemensperger et al., 2005; Sitaraman et al., 2008; Hart et al., 2011; Johnson et al., 2011; Wood et al., 2011; Roberts and Hedlund, 2012).

## Future directions

As the above review indicates, zebrafish larvae possess a surprisingly rich repertoire of learning abilities, including not only non-associative, but also associative and even social learning. Given that at present neurobiologists lack a comprehensive understanding of any form of learning in any organism (discussed in Glanzman, 2009), we should not underestimate the formidable analytic challenge posed by the types of learning and memory that zebrafish larvae are known to exhibit. Moreover, the future is likely to bring an increased appreciation of the cognitive capabilities of these animals, which are almost certainly underrated at present.

In addition to the development of new learning and memory assays in larval zebrafish, one can anticipate that the genetic tools available for use in zebrafish will steadily improve (see Figure 1). At present the most widely used method of altering gene function in larval zebrafish is gene knockdown by morpholinos (Mullins et al., 1994) This method has been widely employed in standard studies of the zebrafish developmental biology, but has had only partial success in behavioral studies due to the temporally restricted nature of the mRNA knockdown (Bill et al., 2009). Many behavioral assays (see, e.g., Valente et al., 2012) require fish to be 5 dpf or older, at which time the efficacy of morpholino gene knockdown is questionable; this method is therefore of limited value in learning and memory research. A relatively new method that should ultimately prove more useful than morpholinos for molecular analyses of behavior is Targeting Induced Local Lesions IN Genomes, (TILLING); here, zebrafish mutants are initially produced by exposure of embryos to the mutagen *N*-ethyl-*N*-nitrosourea (ENU), and then the DNA of these fish is screened and sequenced to identify mutations within specific genes (Wienholds et al., 2003). Currently, the Sanger Institute and the Zebrafish TILLING Project have available a large number of mutant fish with predicted mutations that should code for nonfunctional proteins (http://www.sanger.ac.uk/Projects/D_rerio/zmp/ and http://webapps.fhcrc.org/science/tilling/index.php). This resource should lead to the identification of novel molecular pathways involved in learning and memory. In addition, newer techniques, such as zinc finger nucleases, CRISPR, and TALENS, allow for site-directed mutagenesis of zebrafish genes (Doyon et al., 2008; Meng et al., 2008; Bedell et al., 2012; Dahlem et al., 2012; Moore et al., 2012; Hwang et al., 2013). This ability, available in mice for decades through homologous recombination (Capecchi, 1989), has facilitated the identification of many of the molecules important for learning and memory (Grant et al., 1992; Silva et al., 1992a,b). Site-directed mutagenesis promises to be equally useful in molecular analyses of zebrafish learning and memory. Finally, a powerful genetic tool, the GAL4/UAS system (Scott, 2009, and above), has only just begun to be used in memory research in larval zebrafish (Lee et al.), but is likely to become increasingly popular. Scott et al. (2007) have developed several GAL4 combined enhancer trap zebrafish lines; these will permit UAS-linked transgenes to be targeted to specific regions or, in some instances, specific cell types of the larval brain. The GAL4/UAS system has been used to great effect in mechanistic studies of *Drosophila* memory (Joiner and Griffith, 1999; Zars et al., 2000a,b; Akalal et al., 2006; Kasuya et al., 2009; Berry et al., 2012), and is likely to prove equally valuable in the analysis of memory in larval zebrafish.

Investigators have long taken advantage of the transparency of larvae to image optical activity in the brain of intact zebrafish using calcium indicator dyes (Fetcho and O'Malley, 1995, 1997; Ritter et al., 2001; Higashijima et al., 2003). However, new improvements in imaging techniques, combined with genetic manipulation, has made this basic methodology increasingly powerful. Rather than having to inject calcium indicator dyes into single neurons, investigators can now express genetically encoded calcium indicators, such as GCamPs (Del Bene et al., 2010), in the zebrafish brain. The indicators may be expressed throughout the brain (Ahrens et al., 2012), or expressed in restricted regions of the brain (Del Bene et al., 2010; Muto et al., 2011). Furthermore, increasingly powerful genetically encoded calcium indicators are being developed (Akerboom et al., 2012) and this, together with improvements in optical techniques, such as two-photon microscopy and, more recently, light-sheet microscopy (Ahrens et al., 2013), make it now feasible to record the activity of more than 80% of the neurons in the larval zebrafish brain at the same time with single-cell resolution (Ahrens et al., 2012). This is a remarkable advance, one that could revolutionize our understanding of the brain circuits generate behaviors and encode learned experiences. Moreover, zebrafish larvae are uniquely suited to take advantage of this new technology.

Another new optical method to which zebrafish larvae are highly amenable is optogenetics. Through the use of light-gated glutamate receptors (Szobota et al., 2007), channelrhodopsin (Douglass et al., 2008; Bundschuh et al., 2012; Fajardo et al., 2013) and halorhodopsin (Arrenberg et al., 2009) one can either excite or inhibit neurons in the intact, behaving fish. Also, the specificity of optical manipulation of neuronal activity can be further refined by means of genetic tools. This technology has already been used for mechanistic studies of behavior in zebrafish larvae, although not yet for studies of learning and memory. However, optogenetic investigations of learning and memory have recently been carried out in mice (Alonso et al., 2012; Liu et al., 2012), and one can anticipate similar studies in zebrafish larvae in the near future.

Mention should also be made of other new methods for manipulating or monitoring neural function in the intact larval zebrafish's brain. For example, GAL4-UAS technology can be used to target the expression of tetanus toxin, which blocks neurotransmitter release, to specific neurons and thereby eliminate their contribution to behavior-related brain activity (Asakawa et al., 2008; Wyart et al., 2009). Furthermore, Schuman and colleagues (Hinz et al., 2011) have recently developed methods for identifying and visualizing newly synthesized proteins in the brain of the intact larval zebrafish. As they point out, this methodology should prove useful for determining the molecules that are important for long-term memory.

## Conclusions

Zebrafish larvae are uniquely adapted to new genetic and optical technologies for studies of behavior. For this reason, it is gratifying that these relatively simple animals possess significant capabilities for learning. In particular, as we have discussed in this review, zebrafish larvae exhibit not only short-term, but also long-term memory, as well as associative and social learning. As neurobiologists increasingly recognize the advantages of zebrafish larvae for investigations of learning and memory, our knowledge of the mnemonic repertoire of these animals will undoubtedly expand. Possibly, studies of this tiny, immature, deceptively humble creature will one day unlock some of the most profound secrets about how brains acquire and store memories.

### Conflict of interest statement

The authors declare that the research was conducted in the absence of any commercial or financial relationships that could be construed as a potential conflict of interest.
